# An Apical Meristem-Targeted *in planta* Transformation Method for the Development of Transgenics in Flax (*Linum usitatissimum*): Optimization and Validation

**DOI:** 10.3389/fpls.2020.562056

**Published:** 2021-01-28

**Authors:** Karthik Kesiraju, Shaily Tyagi, Soumyadeep Mukherjee, Rhitu Rai, Nagendra K. Singh, Rohini Sreevathsa, Prasanta K. Dash

**Affiliations:** ICAR-National Institute for Plant Biotechnology, New Delhi, India

**Keywords:** transgenic flax/linseed, *in planta* transformation, apical meristem, *GUS*, *nptII*, GM crops

## Abstract

Efficient regeneration of explants devoid of intrinsic somaclonal variations is a cardinal step in plant tissue culture, thus, a vital component of transgenic technology. However, recalcitrance of economically important crops to tissue culture-based organogenesis ensues a setback in the use of transgenesis in the genetic engineering of crop plants. The present study developed an optimized, genotype-independent, nonconventional tissue culture-independent *in planta* strategy for the genetic transformation of flax/linseed. This apical meristem-targeted *in planta* transformation protocol will accelerate value addition in the dual purpose industrially important but recalcitrant fiber crop flax/linseed. The study delineated optimization of *Agrobacterium tumefaciens-*mediated transformation and stable T-DNA (pCambia2301:*GUS*:*nptII*) integration in flax. It established successful use of a stringent soilrite-based screening in the presence of 30 mg/L kanamycin for the identification of putative transformants. The amenability, authenticity, and reproducibility of soilrite-based kanamycin screening were further verified at the molecular level by GUS histochemical analysis of T_0_ seedlings, *GUS* and *nptII* gene-specific PCR, genomic Southern hybridization for stable integration of T-DNA, and expression analysis of transgenes by sqRT-PCR. This method resulted in a screening efficiency of 6.05% in the presence of kanamycin, indicating amenability of *in planta* flax transformation. The strategy can be a promising tool for the successful development of transgenics in flax.

## Introduction

Unpredictable climatic vagaries, burgeoning global population, and brimming buffer stocks are major concerns that threaten the sustainability of agriculture across all agro-ecological regions of the world. The current pandemic-influenced lockdown worldwide has made it imperative for all nations to maintain a substantial buffer stock by improvement of agricultural productivity and enhancement of harvest index in all crops. Thus, discovery and fast deployment of new technologies such as marker-assisted selection, genomics and genome engineering through RNAi, gene editing through CRISPR-Cas, and technologies to mitigate a plethora of stress factors to increase yield potential are of paramount importance. Such biotechnological interventions in crop improvement programs have globally resulted in engineering plants for stress management. Cumulative inputs from transgenics and genomics in the identification and introgression of superior alleles toward trait-specific improvement have been accomplished in agriculturally important staple crops such as rice, maize, cotton, mustard, and pigeonpea, while non-staple but commercially important crops like jute and flax are orphaned (Majumder et al., 2020). Flax/linseed (*Linum usitatissimum*), besides its inherent nutraceuticals in seeds, is an industrially important dual-purpose crop grown for its fiber and seed oil (Dash et al., 2017). In keeping with the industrial importance of the crop and its enhancement in the given scenario of climate change, global efforts are being made toward flax improvement (Gupta and Dash, 2017; Dash et al., 2018). As with other crops, effectual utilization of genomics and other omics platforms are being made to identify and prospect genomic resources to accelerate varietal improvement programs (Dash et al., 2014, 2015, 2017; Gupta et al., 2017; Shivaraj et al., 2017) in flax. Nevertheless, reports of successful deployment of these genes in flax are meager, owing to its recalcitrance to regeneration through tissue culture methods.

Successful exploitation of the translational utility of superior alleles requires high throughput methodologies for the development of transgenics. As the single major criterion for effective transgenesis is effective totipotency and regeneration, recalcitrance of flax to tissue culture-based regeneration has been the major bottleneck. Hence, there has always been a dearth of successful flax transformation strategies despite several attempts toward standardization and optimization of regeneration as well as non-tissue culture-based approaches (Lamblin et al., 2007; Beranova et al., 2008; Bleho et al., 2012; Bastaki and Cullis, 2014, 2019; Beyaz et al., 2016a,b, 2017; Mandal et al., 2018; Aycan et al., 2019; Majumder et al., 2020).

Cardinal contributions in the field of crop improvement, especially in case of those crops that are difficult to regenerate, have been devised by deploying tissue culture independent *in planta* transformation methodologies (Kesiraju and Sreevathsa, 2017; Karthik et al., 2020). Innumerable strategies that target T-DNA to discrete parts of plants and avoid tissue culture-induced somaclonal variations have emerged to aid genetic engineering of crop plants (Kaur and Sugani, 2019). One such methodology is an apical meristem-targeted *in planta* transformation protocol that targets T-DNA to the growing shoot apical meristem *in vitro* and allows the development of plants *ex vitro* (Singh et al., 2018). Amenability of this approach has been successfully demonstrated to be genotype-independent in an array of dicot (Ramkumar et al., 2019) and monocot (Hatzade et al., 2019) plants. The strategy has aided successful development of transgenic plants imparting protection against drought (Ramu et al., 2016), salinity (Prashantkumar et al., 2011), heavy metal stress (Nagaveni et al., 2011), insects (Singh et al., 2018), and fungal (Sundaresha et al., 2016) diseases.

In this study, we present successful optimization of the apical meristem-targeted *in planta* transformation. Our successful demonstration of a non-tissue culture-based methodology targeting the apical meristem to develop genetically modified flax can be a *bona fide* approach to be incorporated in varietal improvement programs in flax through genetic modification.

## Materials and Methods

### Plant Material and Binary Vector Used for Transformation

**FIGURE 1:**
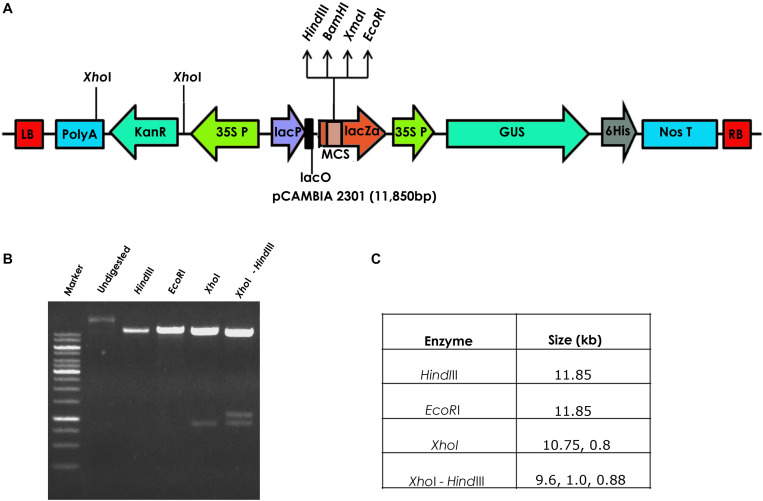
**(A)** Schematic representation of the pCAMBIA2301 vector used for the development of transgenic flax, **(B)** restriction analysis of pCAMBIA2301, and **(C)** details of the restriction profile of pCAMBIA2301.

Seeds of a popular flax cultivar T-397 were surface-sterilized with 1% bavastin for 5 min, washed repeatedly for 3–4 times with distilled water and soaked for 4 h. The seeds were later transferred to Petri dishes and maintained in an incubator at 28°C for 2 days until radicle emergence. *Agrobacterium tumefaciens* strain EHA 105, harboring the binary vector pCambia2301^1^ containing *GUS* gene driven by CaMV35S promoter and *nptII* gene driven by CaMV35S promoter as the plant marker gene was used for flax transformation (Figure 1).

### *Agrobacterium-*Mediated *in planta* Transformation in Flax

Axenic culture of *Agrobacterium* harboring 35S:: *GUS* and 35S:: *nptII* in pCambia2301 from a freshly streaked culture plate was inoculated into 5 ml LB medium (pH 7.0) containing 50 μg/ml kanamycin, 10 μg/ml rifampicin and incubated overnight at 28°C. Further, 3 ml of the starter culture was inoculated into 50 ml of Winans, AB medium (pH 5.2) and incubated for 18 h 28°C at 220 rpm. Two days old germinating flax seedlings with emerging plumule were punctured 3–4 times with an insulin syringe at the apical meristem and incubated in AB minimal medium supplemented with wounded tobacco leaf extract (Rohini and Rao, 2000) at 28°C at 50 rpm for 1 h. The plants were later allowed to grow under optimal conditions (Rohini and Rao, 2000).

### GUS Histochemical Analysis

GUS expression in primary transformants *vis-à-vis* wild type, was assayed 36 h after *Agrobacterium* infection. Seedlings (wild type and infected) were transferred into falcon tubes and incubated overnight with GUS assay buffer (containing 0.1 M phosphate buffer, pH 7.0, 2 mM X-Gluc, 5 mM each of potassium ferricyanide and potassium ferrocyanide and 0.1% Triton X-100) in a 37°C water bath. Seedlings were later decolorized with 75% ethanol and scored for blue coloration in the tissues (Keshamma et al., 2008).

### Kanamycin Screening for the Identification of Putative Transformants

Flax seeds of different T_0_ plants were soaked overnight in distilled water, incubated in 30 mg/L kanamycin solution for 5 h at 28°C with constant agitation of 50 rpm and initially transferred to Petri plates containing soaked filter paper disks. The seeds were later transferred to soilrite in cup trays and allowed to grow in the net-house. Additionally, untreated control (wild type seeds treated with water) and treated control (wild type seeds treated with kanamycin) were grown in different cup trays to compare the effect of kanamycin. After 8–10 days, plants that could grow normally in the presence of kanamycin were selected and transferred to pots supplemented with $\frac{1}{4}$ strength Hoagland solution. Well-established plants were labeled as T_1_ generation transgenics and further analyzed for the presence of transgenes.

### Molecular Analysis of the Identified Transgenic Plants

Young leaves of putative transgenic and wild type flax plants were collected and ground in liquid nitrogen. The genomic DNA from leaf samples was extracted following a modified cetyltrimethyl ammonium bromide (C-TAB) method (Porebski et al., 1997).

### PCR Analysis

PCR reaction mixture (25 μL) consisted of 2.5 μL of 10× Taq buffer, 10 pM each of forward and reverse primer, 200 μM dNTPs, 1 U of Taq DNA polymerase and 100 ng of genomic DNA, made up to a final volume of 25 μL with nuclease-free water was used to amplify the two transgenes viz. *nptII* and *GUS* (Table 1). PCR amplification was carried out in a thermal cycler programmed with initial denaturation at 95°C for 5 min followed by 35 cycles of denaturation at 95°C for 1 min, annealing at 58°C for 1 min for *nptII* gene primers and 55°C for 1 min for *GUS* gene primers and extension at 72°C for 1 min. Final extension was carried out at 72°C for 10 min to amplify specific gene products. While “Blank” consisted of nuclease-free water instead of genomic DNA, wild type contained 100 ng wild type genomic DNA and positive control contained 25 ng pCambia 2301 plasmid. The amplified gene products of 750 bp *nptII* gene and 1 kb *GUS* gene were analyzed on a 0.8% agarose gel.

**TABLE 1:** List of primers used in the study.

Primer ID	Primer sequence (5’–3’)
*NptII* FP *NptII* RP	CCGGAATTCATGATTGAACAA CCCAAGCTTCAGAAGAACTC
*GUS* FP *GUS* RP	TTA TGC GGG CAA CGT CTG GTAT TGA CAA AAA CCA CCC AAG CGT
*NptII* sqRT-FP	ATTGCACGCAGGTTCTCC
*NptII* sqRT-RP	TGTCTGTTGTGCCCAGTCA
*GUS* sqRT-FP *GUS* sqRT-FP	ACCTCGCATTACCCTTACGCTG CCCGCTTCGAAACCAATG

### Genomic Southern Analysis

In order to determine the T-DNA copy number in transgenic plants, 15 μg of genomic DNA each from putative flax transgenics and wild type plant was digested with *Hind*III (overnight) and separated on a 0.8% agarose gel. The separated fragments were later transferred to positively charged nylon membrane by capillary transfer using 20× SSC. The membrane was hybridized with a DIG labeled 750 bp *nptII* gene fragment prepared using DIG Oligonucleotide 3’-End Labeling Kit. Hybridization and subsequent washings were carried out according to manufacturer’s instructions (Roche, Detection of digoxigenin-labeled nucleic acids by enzyme immunoassay and enzyme-catalyzed color reaction with NBT/BCIP, Roche Holding AG, Basel, Switzerland). The membrane was exposed to NBT/BCIP solution and observed for gene-specific bands to determine the copy number in the transgenic plants.

### Analysis of Transgenic Plants for Transcript Accumulation by sqRT-PCR

Total RNA was isolated from transgenic and wild type flax plants using a total RNA isolation kit (Spectrum^TM^, Sigma-Aldrich, St. Louis, MO, United States) and quantified. cDNA was synthesized from 2 μg total RNA according to manufacturer’s instructions (SuperScript^®^ VILO^TM^, Invitrogen, Carlsbad, CA, United States) and used for cDNA preparation. To evaluate transcript accumulation, 1 μl of diluted cDNA mix was used as a template for the amplification of 67 bp *nptII* and 111 bp *GUS* fragments (Table 1). The PCR reaction mixture (25 μL) consisted of 2.5 μL 10× Taq buffer, 10 pM each of forward and reverse primer, 200 μM dNTPs, and 1 U of Taq DNA polymerase (Bangalore Genei, Bengaluru, India); 1 μL of cDNA was made up to a final volume of 25 μL with nuclease-free water. PCR amplification was carried out in a thermal cycler (Eppendorf, Hamburg, Germany) programmed with initial denaturation at 95°C for 4 min followed by 30 cycles of denaturation at 95°C for 30 s, annealing at 58°C for 30 s (for both *nptII* and *GUS* gene RT-primers, Table 1) and extension at 72°C for 30 s. The final extension was carried out at 72°C for 7 min to amplify specific gene products. “Blank” consisted of nuclease-free water instead of cDNA, wild type contained 1 μL of cDNA of wild type, and positive control contained 25 ng of pCambia 2301 plasmid. The amplified gene products were analyzed on a 2.0% agarose gel.

## Results and Discussion

*In planta* transformation strategies aid in the development of transgenic plants by completely circumventing tissue culture steps and have cardinal contributions in plant biotechnology (Kesiraju and Sreevathsa, 2017; Karthik et al., 2020). Despite efforts toward demonstration of the non-tissue culture based transformation in flax (Bastaki and Cullis, 2014; Maher et al., 2020; Majumder et al., 2020; Tyagi et al., 2020; Zlobin et al., 2020), development of large-scale stable transgenics for functional genomics study in flax is still lacking. The present study provides proof for the optimization of an apical meristem-targeted transformation strategy in flax and, hitherto, development of stable transgenics.

### *Agrobacteium tumefaciens-*Mediated *in planta* Transformation of Flax

**FIGURE 2:**
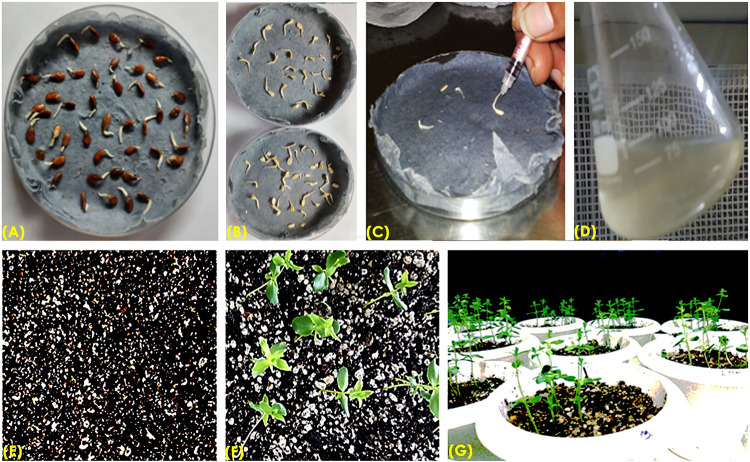
Overview of apical meristem targeted *in planta* transformation strategy for the development of transgenics in flax. **(A)** Immersed seeds for germination, **(B)** removal of seed coat of immersed seeds to facilitate *Agrobacterium* infection, **(C)** apical meristematic region of seedling was punctured with an insulin syringe, **(D)** co-cultivation of embryos in AB minimal medium, **(E)** post-infection embryos and soilrite planting, **(F)** recovery of plants on soilrite, and **(G)** establishment of recovered plants in the greenhouse.

The authenticity of pCambia2301 plasmid (Figure 1A) was initially confirmed by restriction digestion using *Hind*III and *Eco*RI enzymes (Figures 1B,C). Optimization of conditions for flax transformation was essentially based on the standardized *in planta* protocol (Rohini and Rao, 2000). Accordingly, various steps of the methodology were optimized for co-cultivation and efficient infection (Figure 2). About 82 healthy seeds were subjected to transformation by removing the seed coat of 2-day-old germinated seeds. The emerging plumule was punctured at the apical region (Figures 2A–C) and co-cultivated in AB minimal media containing wounded tobacco leaf extract that activates virulence for enhanced infectivity of *Agrobacterium* (Figure 2D). The seedlings after infection were repeatedly washed with distilled water, transferred to Petri dishes containing filter paper disks soaked with distilled water and placed in dark overnight. The next day, seeds were transferred to pots containing autoclaved soilrite saturated with Hoagland’s solution and maintained in growth chambers at 25°C with 16 h light and 8 h dark photoperiod (Figures 2E–G). It was observed that the growth of infected seedlings was slow when compared with uninfected wild type.

### Preliminary Screening of Transformants

Amenability of primary transformants of any plant species to the *in planta* transformation protocol have been assessed with the help of screenable markers (Kumar et al., 2009). Expression of scorable marker genes like *GUS* and *GFP* usually provide early indications of transformability and/or successful transformation of infected tissues. Therefore, in the present study, GUS histochemical analysis of the primary transformants was used as an initial proof of transformation.

**FIGURE 3:**
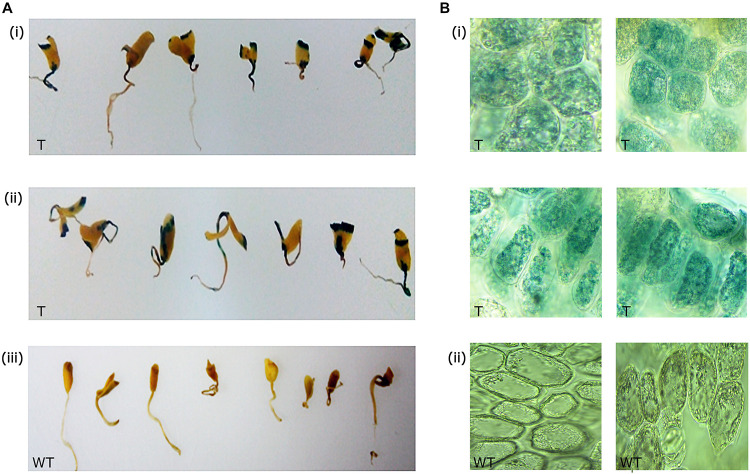
*GUS* expression in primary transformants. **(A)** *GUS* expression in the shoot apical region of **(i,ii)** primary transformants, and **(iii)** wild type, **(B)** sections of the apical meristematic region showing GUS expression within cells in the primary transformants **(i)**, and sections of wild type **(ii)** showing absence of GUS expression.

Out of 82 primary transformants, GUS histochemical analysis was performed using 50 seedlings, and the remaining seedlings were allowed to recover and grow into healthy plants. It was observed that nearly 32 seedlings showed GUS expression (blue spots) at the site of infection (Figures 3Ai,ii). Wild type seedlings remained colorless as they lacked the *GUS* transgene (Figure 3Aiii). Sections of the GUS-stained tissues further confirmed the expression of *GUS* within the cells (Figures 3Bi,ii). Approximately 64% of the total seedlings analyzed showed *GUS* expression at the infection site indicating infection of *Agrobacterium* as a chance event that depended on explant and tissue type.

The presence of the scorable *GUS* marker gene in transgenic flax made it feasible to investigate the suitability of apical meristem-targeted *in planta* transformation for development of transgenic flax. Based on the initial leads of transformability in flax, the remaining 32 plants were allowed to recover and grow into healthy plants in the greenhouse.

### Recovery of Primary Transformants

**FIGURE 4:**
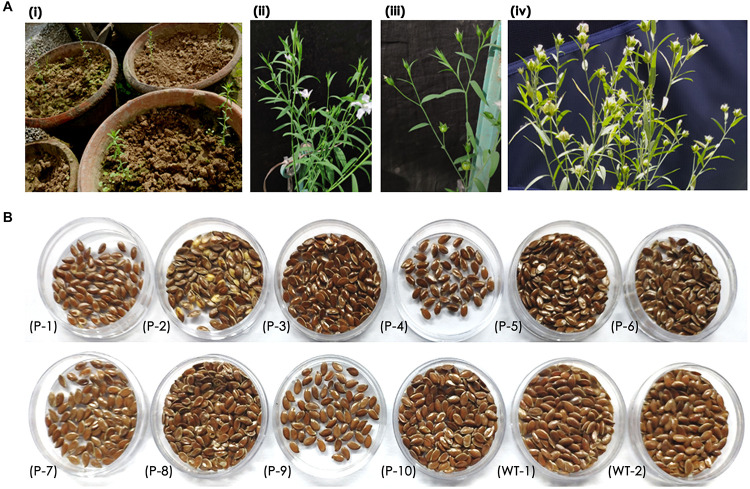
**(A)** Recovery of primary transformants in the greenhouse, **(i)** transfer of healthy plants to pots, **(ii,iii)** transgenic plant showing normal growth, flowering, and bud set, **(iv)** transgenic plant with mature capsules, and **(B)** an overview of seed pool collected from 10 transgenic and wild type plants.

Recovery of seedlings after *in planta* transformation and establishment of primary transformants is an important step in the development of stable transgenics. The percentage of recovery in most plants depends on the response of plants to infection stress as well as successful hardening. In the present study, 15 of 32 plants that were transferred to greenhouse could survive. These plants were transferred to pots filled with soil and grown in the greenhouse (Figure 4Ai). However, 10 plants bloomed and set capsules with seeds (Figure 4Aii,iii). The seed pool was uniform but varied in the number of seeds per capsule (Figure 4B) as the growth and vigor of transgenic plants was slow in comparison to wild type plants.

It was observed that the recovery of transformants in flax was low as a total of 10 plants survived mechanical injury, infection, and hardening stress to complete their life cycle and set seeds. Nonetheless, a recovery efficiency of 3.2% was achieved after transformation, possibly due to the slender seedling phenotype.

### Analysis of the T_1_ Generation Plants for the Identification of Putative Transformants

*In planta* transformation of the shoot apical meristem targets T-DNA to the differentiating cells and results in T_0_ plants that are chimeric in nature (Rohini and Rao, 2000). Hence, identification of putative transformants in principle is carried out in the T_1_ generation. By and large, any type of transformation strategy involves the use of selectable marker genes like herbicides and antibiotics for segregation of transformed plants from the others (Shivakumara et al., 2017; Singh et al., 2018). However, studies in flax as well as in other crops have demonstrated the use of non-selectable marker-based screening such as grid PCR (Keshamma et al., 2008; Kumar et al., 2009; Sundaresha et al., 2010), direct PCR (Kumar et al., 2009; Bastaki and Cullis, 2014), and dot blot Southern analysis (Kumar et al., 2011) for distinguishing putative transformants. However, stringent and robust screening using concentrations of selection agents deleterious to wild type plants have been an authentic method of identification of transformed plants (Singh et al., 2018).

**FIGURE 5:**
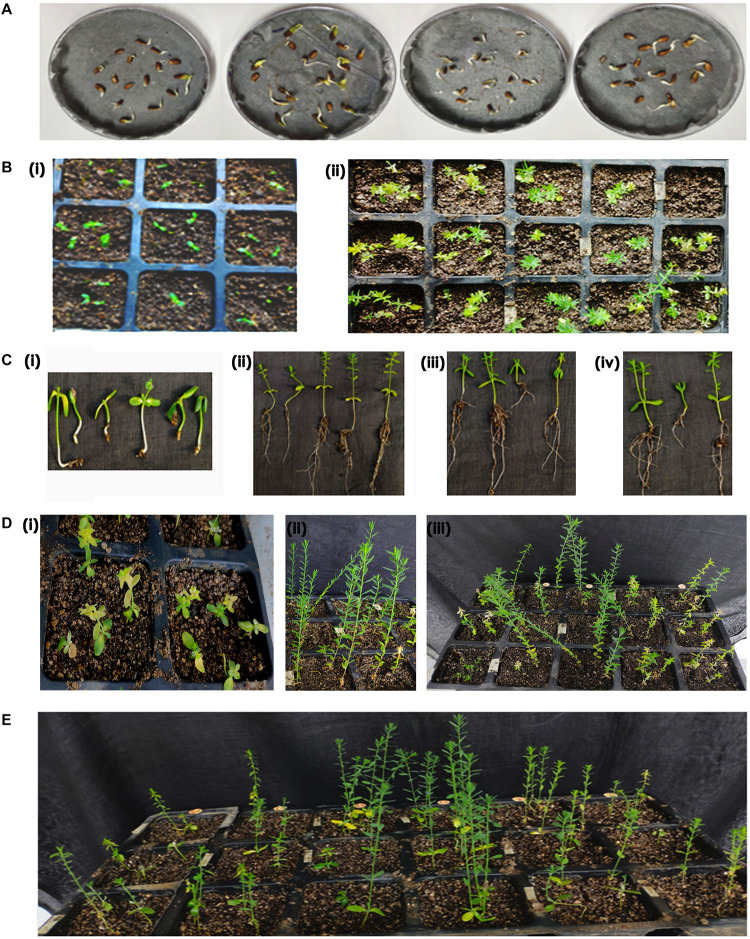
Screening of putative transformants under kanamycin selection. **(A)** Transfer of seedlings of independent T_0_ plants after kanamycin treatment, **(B)** cup tray screening for antibiotic resistance, **(i)** overview of cup tray with planted seeds, **(ii)** initial signs of kanamycin induced necrosis after 4^th^ day of treatment, **(C)** variation in root and shoot lengths of plants after 10^th^ day of kanamycin treatment, **(i)** treated wild type, **(ii)** untreated wild type, **(iii,iv)** transgenic plants with phenotype at par with untreated wild type, **(D)** an overview of **(i)** treated wild type plants exhibiting severe necrosis, **(ii)** untreated wild type, **(iii)** putative transgenics, and **(E)** an overview of the variable response in T_1_ seedlings to kanamycin treatment exhibiting resistance and susceptibility to kanamycin.

**FIGURE 6:**
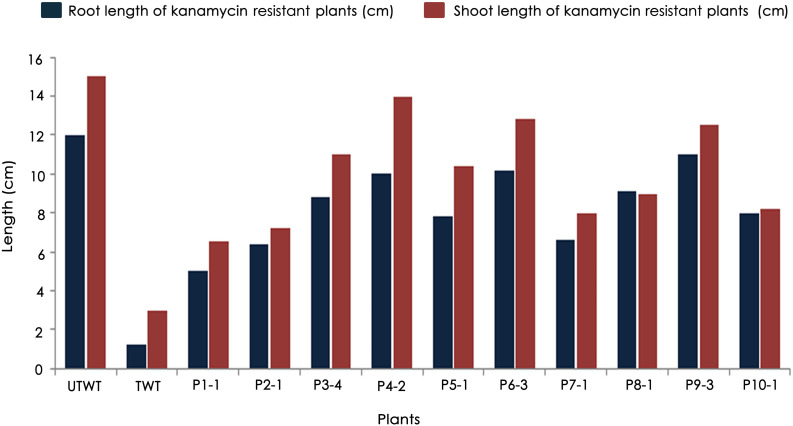
Variations in root and shoot lengths of wild type (untreated and treated) and T_1_ generation transgenic plants in response to 30 mg/L kanamycin.

Accordingly, putative transformants of flax in the present study were identified using a high throughput soilrite-based screening methodology under kanamycin selection pressure. About 40 seeds from each of the 10 surviving primary transformants (T_0_) of flax along with wild type were used for kanamycin screening. Overnight imbibed seeds were treated with 30 mg/L kanamycin solution and transferred to Petri plates containing filter paper disks soaked in water (Figure 5A). Two days later, the seeds were planted in cup trays, allowed to grow (Figure 5Bi), and observed at regular intervals. After 4 days, symptoms of kanamycin stress were visible in all plants that were being screened including treated wild type plants (Figure 5Bii). After 10 days, variations in root and shoot length were observed in treated wild type (Figure 5Ci), untreated wild type (Figure 5Cii), and transgenics (Figures 5Ciii,iv). While treated wild type plants exhibited severe necrotic symptoms and retarded growth (Figure 5Di), untreated wild type plants were healthy as they did not receive kanamycin treatment (Figure 5Dii). However, the T_1_ generation seedlings exhibited variation in their response to kanamycin treatment. While some plants exhibited healthy growth, some exhibited necrotic symptoms (Figure 5Diii). By end of the 10^th^ day of kanamycin treatment, conspicuous morphological variation across the seedlings (Figure 5E) subjected to screening could be seen with the kanamycin-resistant plants easily distinguishable from susceptible plants. Plants exhibiting necrosis were discarded and healthy plants were shifted to green house. As a result, out of 400 transgenic seeds taken for screening, 26 plants recovered, accounting for 6.05% of the total seedlings screened (Table 2). The plants that could resist kanamycin selection displayed growth at par with the untreated control (Figure 6, a representative portrayal of root and shoot length of selected seedlings). These were tagged and grown in the greenhouse as T_1_ generation plants but considered as putative transformants. Earlier studies in the development and analysis of transformants in flax expressed concern over the use of antibiotic selection in the regeneration medium for the identification of transformants due to either escapes or false positives and reduced efficiency of transformation (Bastaki and Cullis, 2014). This soilrite-based screening strategy in flax, however, proves that our screening strategy was not only a stringent method for selection of transgenics but also a robust technique carried out under natural conditions to reduce escapes.

**TABLE 2:** Percentage of T_1_ plants resistant to 30 μg/L kanamycin.

Plant id	No. of seeds taken for screening	No. of seeds resistant to kanamycin	No. of healthy plants after screening	% of plants recovered from kanamycin stress
UT WT	40	NA	NA	NA
T WT	40	0	0	0
P-1	40	2	1	0.4
P-2	40	3	2	0.8
P-3	40	5	5	2
P-4	40	10	5	2
P-5	40	4	2	0.8
P-6	40	8	4	1.6
P-7	40	2	1	0.4
P-8	40	2	1	0.4
P-9	40	7	4	1.6
P-10	40	3	1	0.4
Total	400	46	26	

### Molecular Characterization of Transgene Integration in Flax

**FIGURE 7:**
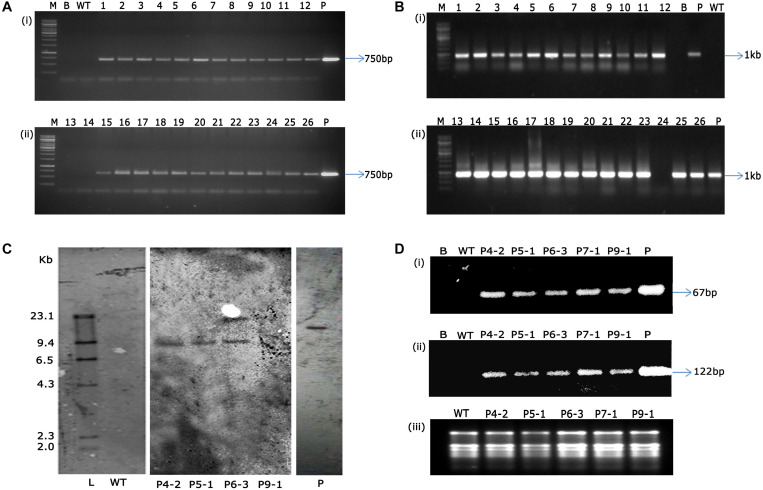
PCR analysis of T_1_ transgenic plants and wild type plants showing amplification of 750 bp *nptII* gene **(A)** and 1 kb *GUS* gene **(B)** [Lane M: 1 kb marker (Thermo scientific), Lane B: water blank (all PCR components except template DNA), Lane WT: wild type plant, Lanes 1-26: putative T_1_ transgenic plants obtained after kanamycin screening, Lane P: binary vector pCambia2301 as positive control], **(C)** genomic Southern analysis of transgenic plants probed with DIG-labeled 750 bp *nptII* gene fragment, Lane L: Lambda *Hind*III DNA digest, Lane WT: wild type, Lanes 1–4: transgenic plants (P4-2, P5-1, P6-3, P9-1, respectively), Lane P: linearized plasmid of pCambia 2301, **(D)** sqRT-PCR analysis for the assessment of transcript accumulation of **(i)** *nptII*, **(ii)** *GUS* genes, and **(iii)** total RNA as loading control. [Lane B: water blank (all PCR components except template DNA), Lane WT: wild type plant, and transgenic plants P4-2, P5-1, P6-3, P7-1, P9-1, Lane P: binary vector pCambia2301 as positive control].

Authentication of transgene integration in 26 (T_1_ generation) flax plants was carried out by PCR analysis using *nptII* and *GUS* gene-specific primers. While all the 26 transgenic plants showed amplification of 750 bp *nptII* and 1 kb fragment of *GUS* gene (Figures 7A,B), wild type plant DNA did not show any amplification by PCR assay. This not only confirmed the presence of transgenes but validated the reproducibility and robustness of soilrite-based kanamycin screening in identification of transformants in flax.

Several studies have been attempted globally to demonstrate transformability of flax using diverse strategies (Beranova et al., 2008; Bastaki and Cullis, 2014; Mandal et al., 2018; Majumder et al., 2020). However, demonstration of stable transgene integration through genomic Southern analysis was underprovided in those studies. In the present study, we have demonstrated that the shoot apical meristem-targeted *in planta* transformation not only introduced kanamycin resistance in flax but also had the stability of gene introgression in the flax genome. Precise endorsement of T-DNA integration was established in three transgenic flax events, P4-2, P5-1, and P6-3. The variation in the number and pattern of bands identified the events to have the transgene stably integrated, demonstrating the independent nature of the transgenic plants. It was observed that events P4-2 and P5-1 displayed single-copy integration of the transgene while event P6-3 had two copies of the transgene integrated in its genome (Figure 7C). Further, accumulation of transcripts as provided by sqRT-PCR confirmed the stable integration and expression of transgenes in the selected transgenic plants (Figures 7Di–iii) *vis-à-vis* their absence in the wild type plants.

Transgenic technology has emerged as an indispensable component of crop improvement programs worldwide. High throughput transformation and screening protocols are of great importance for successful biotechnological intervention. This methodology of *in planta* transformation, which is the first demonstration of stable transgenic development in flax, is a laudable contribution in the field of crop biotechnology. Such a gene transfer methodology holds promise for flax varietal improvement programs.

## Data Availability Statement

The raw data supporting the conclusions of this article will be made available by the authors, without undue reservation.

## Author Contributions

KK, RS, and PKD designed the experiments. KK developed the transgenic plants and identified the putative transformants. KK, ST, and SM performed the molecular analyses. KK, RS, and PKD wrote the manuscript. RR and NS gave input and critically edited the manuscript. PKD and RR were responsible for fund acquisition. All authors contributed to the article and approved the submitted version.

## Conflict of Interest

The authors declare that the research was conducted in the absence of any commercial or financial relationships that could be construed as a potential conflict of interest.

## Footnotes

1 www.cambia.org
